# HDAC8 Activates AKT through Upregulating PLCB1 and Suppressing DESC1 Expression in MEK1/2 Inhibition-Resistant Cells

**DOI:** 10.3390/cells10051101

**Published:** 2021-05-04

**Authors:** Soon-Duck Ha, Naomi Lewin, Shawn S. C. Li, Sung-Ouk Kim

**Affiliations:** 1 Department of Microbiology & Immunology, Schulich School of Medicine & Dentistry, University of Western Ontario, London, ON N6G 2V4, Canada; sha3@uwo.ca (S.-D.H.); naomi.lewin@mail.utoronto.ca (N.L.); 2 Department of Biochemistry, Schulich School of Medicine & Dentistry, University of Western Ontario, London, ON N6G 2V4, Canada; sli@uwo.ca

**Keywords:** lethal toxin, AKT, PLCB1, PI-PLC, DESC1, HT-29 cells, cancer, resistance

## Abstract

Inhibition of the RAF-MEK1/2-ERK signaling pathway is an ideal strategy for treating cancers with NRAS or BRAF mutations. However, the development of resistance due to incomplete inhibition of the pathway and activation of compensatory cell proliferation pathways is a major impediment of the targeted therapy. The anthrax lethal toxin (LT), which cleaves and inactivates MEKs, is a modifiable biomolecule that can be delivered selectively to tumor cells and potently kills various tumor cells. However, resistance to LT and the mechanism involved are yet to be explored. Here, we show that LT, through inhibiting MEK1/2-ERK activation, inhibits the proliferation of cancer cells with NRAS/BRAF mutations. Among them, the human colorectal tumor HT-29 and murine melanoma B16-BL6 cells developed resistance to LT in 2 to 3 days of treatment. These resistant cells activated AKT through a histone deacetylase (HDAC) 8-dependent pathway. Using an Affymetrix microarray, followed by qPCR validation, we identified that the differential expression of the phospholipase C-β1 (PLCB1) and squamous cell carcinoma-1 (DESC1) played an important role in HDAC8-mediated AKT activation and resistance to MEK1/2-ERK inhibition. By using inhibitors, small interference RNAs and/or expression vectors, we found that the inhibition of HDAC8 suppressed PLCB1 expression and induced DESC1 expression in the resistant cells, which led to the inhibition of AKT and re-sensitization to LT and MEK1/2 inhibition. These results suggest that targeting PLCB1 and DESC1 is a novel strategy for inhibiting the resistance to MEK1/2 inhibition.

## 1. Introduction

Hyperactivation of the MEK1/2-ERK signaling axis due to mutations in NRAS and BRAF drives oncogenesis in ~30% of human cancers, and targeting RAF and MEK can be a curative therapy for these cancers [1]. However, the development of resistance often prompts clinical relapse and therapeutic failure. Among various causes, incomplete inhibition of the MEK1/2-ERK pathway contributes to be an intrinsic and acquired resistance to these inhibitors [2,3]. Indeed, combinatory therapies using both RAF and MEK1/2 inhibitors provide a better prognosis and are the current standard-of-care in certain cancers [4,5]. The anthrax lethal toxin (LT), which potently inhibits MEK1/2-ERK activation and can be modified to selectively target cancers, is a promising biomolecule [6,7,8], likely with less of a chance of resistance development. LT, which is composed of a carrier protective antigen (PA) and protease lethal factor (LF), selectively cleaves the N-termini of all MEKs, except MEK5 [9,10], and induces cell cycle arrest and cell death [11,12]. However, we showed that macrophages adaptively respond to LT and become resistant to LT-induced cell cycle arrest through activating the phosphatidylinositol 3-kinase (PI3K)/AKT signaling cascade [13,14]. Similarly, in certain tumor cells, resistance to RAF/MEK inhibitors is attributed to activation of the PI3K-AKT signaling axis caused by a loss of phosphatase and tensin homology (PTEN) or adaptive stress responses [15,16,17]. However, the mechanisms that activate the PI3K-AKT signaling pathway in resistant cancer cells are yet to be fully delineated. As one of the potential mechanisms, we previously showed that histone deacetylase 8 (HDAC8), which is a member of the class I HDAC family, is involved in the resistance to LT in macrophages [14]. HDAC8 was also shown to mediate the resistance to RAF inhibitors in melanoma [18]. In these cells, HDAC8 deacetylates and activates the c-JUN transcription factor, resulting in the increased expression of receptor tyrosine kinases and ERK activation. Therefore, HDAC8 may induce a resistance to RAF-MEK inhibition in different pathways, depending on the cell type. To further delineate the mechanisms of HDAC8 in resistance to MEK1/2-ERK inhibition, we examined whether LT induces resistance and, if so, then what mechanisms are involved in cancer cell types with known mutations in the RAS-RAF-MEK signaling axis. We found that HDAC8 was required for a resistance to LT and the MEK1/2 inhibitor U0126 in the human colorectal tumor cell line HT-29 and murine melanoma B16-BL6 cells. HDAC8 induced AKT activation in these resistant cells, in part, through inducing PLCB1 expression. The inhibition of HDAC8 suppressed PLCB1 expression but enhanced DESC1 expression, both of which were involved in preventing the compensatory activation of AKT and resistance to MEK1/2 inhibition.

## 2. Materials and Methods

Reagents—Protective antigen (PA) and lethal factor (LF) were purchased from the List Biological Laboratories (Campbell, CA, USA). The ERK inhibitor U0126, p38 MAPK inhibitor SB203580, AKT inhibitor, and MTT (3-(4,5-dimethylthiazol-2-yl)-2,5-diphenyltetrazolium bromide) were obtained from APExBIO Technology (Houston, TX, USA), Selleck Chemicals (Houston, TX, USA), Calbiochem (San Diego, CA, USA), and Sigma-Aldrich (St. Louis, MO, USA), respectively. HDAC8 inhibitor PCI-34051, edelfosine, and 4-(2-Aminoethyl)benzenesulfonyl fluoride hydrochloride (AEBSF) were obtained Cayman Chemical (Ann Arbor, MI, USA). The HDAC8 and PLCB1 antibodies were obtained from AB clonal Technology (Woburn, MA, USA). Antibodies for phospho-AKT (Ser-473), MEK1 (N-terminal 12 amino acids), and β-actin were purchased from Cell Signaling (Danvers, MA, USA), Stressgen Biotechnologies (Cat# KAP-MA010; Ann Arbor, MI, USA), and Rockland Inc. (Gilvertsville, PA, USA), respectively. The cOmpleteTM EDTA-free protease inhibitor cocktail and phosphatase inhibitor cocktail (phosSTOP) tablets were obtained through Thermo Scientific (Roche; Indianapolis, IN, USA). DESC1 (vector ID; VB170123-1118ntk, hTMPRSS1 (ORF023752)) plasmid was constructed through Cyagen (Vector Builder; Chicago, IL, USA).

Cell culture—Mouse B16-BL6 melanoma, human colorectal tumor HT-29 cells, and human melanoma MDA-435 and SK-MEL-5 cells were maintained in complete RPMI 1640 or DMEM, supplemented with 10% heated-inactivated fetal bovine serum (WISENT; Saint-Jean-Baptiste, QC, Canada, 10-mM MEM nonessential amino acid solution, 100-U/mL penicillin G sodium, 100-μg/mL streptomycin sulfate, and 1-mM sodium pyruvate.

Cell viability and proliferation assay—Cell viability/proliferation was measured by the MTT analysis, as previously described [13]. Briefly, cells were seeded in 96-well plates and cultured in the presence or absence of LT (LF and PA) and/or chemical inhibitors for the time indicated. MTT at a final concentration of 0.5 mg/mL was added and incubated 2–4 h before stopping the experiments by replacing the cell culture media with 100 µL of dimethyl sulfoxide to dissolve the crystals. For cells in suspension, the experiments were ended by adding 100 µL of 0.04-N HCl in isopropanol for 30 min in a shaker at room temperature. Optical densities of each well were analyzed using an automatic microplate reader (Synergy H4 Hybrid Reader, BioTek; Winooski, VT, USA) at a wavelength of 570 nm. The % of cell survival was calculated based on cell numbers in comparison with those of nontreated cells. The % of cell proliferation was based on the cell numbers in comparison with those of nontreated cells 24 h after seeding cells. All cell numbers were estimated based on the standard curve generated by optical densities of known cell numbers.

Gene expression microarray—HT-29 (3 × 10^6^) cells were cultured with or without LT (500 ng/mL of each PA and LF) or LT+ PCI-34051 (PCI: 5 μM) for 48 h. Total cellular RNAs were prepared using TRIzol^TM^ (Ambion Inc.; Carlsbad, CA, USA), and the quantity and quality of the total RNAs were verified through an Agilent 2100 Bioanalyzer. Total RNAs (100 ng) were then amplified and labeled to prepare complementary RNAs, 5.5 µg of which was loaded onto the array, following the manufacturer’s guidelines (Affymetrix, Santa Clara, CA, USA). Gene array was performed using the GeneChip™ Human Genome U133 Plus 2.0 Array kit in the London Regional Genomics Centre at Western University, London, ON, Canada. CEL files were then imported to Partek^TM^ Genomics Suite^TM^ for differential gene expression (with 2-fold change cut-off) and gene ontology enrichment analyses.

Immunoblotting—Total cell lysate preparation and Immunoblotting were conducted as previously described [13]. Briefly, cells were lysed in ice-cold lysis buffer (20-mM MOPS, 2-mM EGTA, 5-mM EDTA, 1-mM Na3VO4, 40-mM β-glycerophosphate, 30-mM sodium fluoride, 20-mM sodium pyrophosphate, 0.1% SDS, and 1% Triton X-100, pH 7.2) containing a cOmplete^TM^ EDTA-free protease inhibitor and phosphatase inhibitor (phosSTOP), and the cells were incubated on ice for 10 min. Whole lysates were centrifuged at 12,500 rpm for 15 min at 4 °C. Proteins in supernatants were separated by SDS-polyacrylamide gels and transferred onto a nitrocellulose membrane (Bio-Rad, Hercules, CA, USA). The membranes were blocked with 5% (*w*/*v*) skim milk for 1 h at room temperature and exposed to primary antibodies overnight at room temperature and then washed three times with 1 × TBST (20-mM Tris and 150-mM NaCl, pH 7.5) containing 0.05% Tween 20. The membranes were incubated with the secondary antibody for 60 min at room temperature, and immunoreacting bends were developed using an Enhanced Chemiluminescence detection system (ECL; Thermo Scientific; Rockford, IL, USA) or Luminata^TM^ Forte (Millipore, Billerica, MA, USA).

Quantitative real-time PCR—Quantitative real-time PCR (qPCR) was carried out as previously described [13]. Briefly, the isolation of total cellular RNAs and reverse transcribing were performed using TRIzol^TM^ (Ambion Inc.) and Moloney murine leukemia virus (M-MuLV) reverse transcriptase (New England Biotechnology; Ipswich, MA, USA), according to the manufacturer’s instructions. The qPCR analyses were processed using a Rotor-Gene RG3000 quantitative multiplex PCR instrument (Montreal Biotech Inc.; Dorval, QC, Canada) and Power UP^TM^ SYBR Green Master Mix (Applied Biosystems, Life Technologies; Foster City, CA, USA). Data were normalized to the levels of the glyceraldehyde 3-phosphate dehydrogenase (GAPDH) housekeeping gene. Human primers used for qPCR were: for GAPDH, 5′-ACCCACTCCTCCACCTTTG-3′ (5′ primer) and 5′-CTCTTGTGCTCTTGCTGGG-3′ (3′ primer); for HDAC8, 5′-ATTCTCTACGTGGATTTGGATC-3′ (5′ primer) and 5′-ATGCCATCCTGAATGGGCACA-3′ (3′ primer); for PLCB1, 5′-GGTGCAGTATATCAAGAGGCTAGA-3′ (5′ primer) and 5′- TGGTCACCACTTGAGAGCTT-3′ (3′ primer); and for DESC1, 5′-AGAGTTTGTTGGGAACCCTGG-3′ (5′ primer) and 5′-AAGCCTCTCTGCCAAACTCAG-3′ (3′ primer). Mouse PLCB1 and DESC1 mRNA expression were analyzed using primers GAPDH, 5′-GCATTGTGGAAGGGCTCATG-3′ (5′ primer) and 5′-TTGCTGTTGAAGTCGCAGGAG-3′ (3′ primer); PLCB1, 5′-GGTGCAGTATATCAAGAGGCTAGA-3′ (5′ primer) and 5′-TGGTCACCACTTGAGAGCTT-3′ (3′ primer); and DESC1, 5′-AGAGTTTGTTGGGAACCCTGG-3′ (5′ primer) and 5′-AAGCCTCTCTGCCAAACTCAG-3′ (3′ primer).

Transfection of small interfering (si)RNAs and plasmids—HT-29 cells were transfected with human HDAC8-targeting siRNA [(Invitrogen, Life Technologies; Carlsbad, CA, USA) catalog No. 10620318-19, HDAC8HSS125194)] or human PLCB1-targeting siRNA (Invitrogen, Life Technologies, Cat No. 10620318-329608F08 and 10620319-329974A06, PLCB1VHS41619) at 64 nM for 18 h using Lipofectamine RNAiMAX (Invitrogen, Life Technologies), according to the manufacturer’s instructions. Cells were then replated to 6-well or 96-well plates, and, after incubation for an additional 6 h, cells were treated with LT or U0126 for the time indicated. Plasmid transfection was carried out using Lipofectamine 2000 or 3000 (Invitrogen, Life Technologies) according to the manufacturer’s instructions. Briefly, 1.5 × 10^6^ cells were plated on 6-well plates at 6–8 h prior to transfection and transfected with plasmids for 4 h. Cells were further incubated for 16–18 h with an additional cell culture medium. Cells were then replated and treated with or without LT or U0126 for the time indicated.

Statistical analysis—Data were analyzed using GraphPad Prism Version 4.0 software, and the results were presented as the mean ± SD of two or three independent repeats. Statistical significance was defined as *p* < 0.05 (*).

## 3. Results

### 3.1. Murine Melanoma B16-BL6 and Human Colorectal HT-29 Cells Develop Resistance to LT in an HDAC8-Dependent Pathway

**Figure 1:**
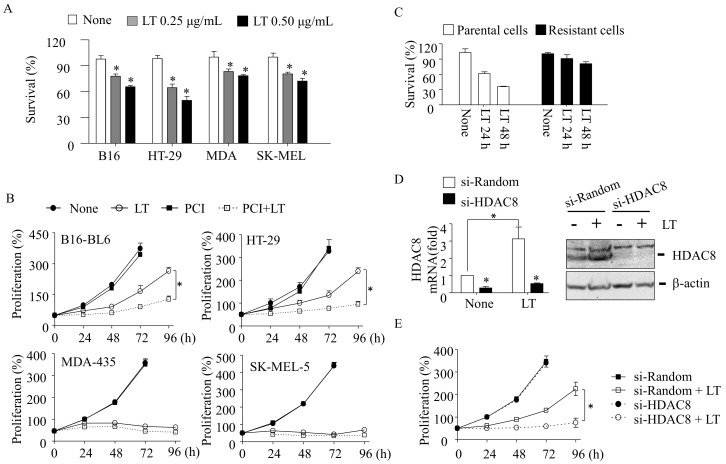
HDAC8 inhibition prevents a resistance to LT-induced cell cycle arrest in B16-BL6 melanoma and HT-29 colorectal cancer cells. (**A**) Cells were seeded in 96-well plates and treated with two different doses of LT (each PA and LF) for 24 h. (**B**) B16-BL6, HT-29, MDA-MB-435, and SK-MEL-5 cells were cultured with LT (500 ng/mL of each PA and LF) in the presence or absence of PCI-34051 (PCI: 1 µM) for the time indicated. (**C**) Resistance HT-29 cells were selected after treating LT for 72 h, and the surviving cells were replated and re-exposed to LT (500 ng/mL of each PA and LF) for the time indicated. (**A**–**C**) Cell survival and proliferation were measured by the MTT assay. (**D**,**E**) HT-29 cells were transfected with random or HDAC8-specific siRNAs (64 nM) for 18 h. Cells were then cultured in the presence or absence of LT (500 ng/mL of each PA and LF) for 48 h (**D**) or for the time indicated (**E**). HDAC8 mRNA and the protein levels were measured by RT-qPCR ((**D**), left panel) and Western blotting using anti-HDAC8 and anti-β-actin (for the loading control) ((**D**), right panel). Cell proliferation was measured by the MTT assay (**E**). (**A**–**E**) Data are expressed as means and ± SD (*n* ≥ 3; *, *p* < 0.05 by the Student’s *t*-test).

We first examined the cytotoxic effect of LT in four cancer cell lines with known mutations in the signaling axis of RAS-RAF-MEK1/2: murine melanoma B16-BL6, human colorectal cancer HT-29, and human melanoma MDA-MB-435 (MDA) [19] and SK-MEL-5 (SK-MEL) cell lines [6,20,21,22,23]. LT was able to decrease cell survival within 24 h in these cells (Figure 1A). To further examine whether the decreased cell survival was due to cell death or cell cycle arrest, small numbers of these cells were plated and monitored for cell proliferation over 96 h in the presence of LT. During the 48 h of LT treatment, no apparent changes in live cell numbers were detected (Figure 1B). In 72 h of LT treatment, the live cell numbers of B16-BL6 and HT-29 cells started increasing, whereas those of MDA and SK-MEL cells did not. These results suggest that B16-BL6 and HT-29 cells became resistant to LT and started proliferating in the presence of LT. Since HDAC8 plays a key role in the resistance to LT cytotoxicity in macrophages [14], we examined if HDAC8 was also involved in the resistance in B16-BL6 and HT-29 cells. As in macrophages, both B16-BL6 and HT-29 cells failed to proliferate in the presence of LT when the cells were treated together with the HDAC8-specific inhibitor PCI34051 (PCI; Figure 1B). PCI alone had no apparent effect on the cell proliferation. To further confirm that the proliferating cells in the presence of LT are resistant cells, rather than cells delayed in cell proliferation, these cells were replated and rechallenged with LT and examined for cell survival. Unlike in parental cells, these resistant cells did not show any defects in cell survival/proliferation (Figure 1C). To further confirm the role of HDAC8, HDAC8 was knocked down by small interference (si)RNAs in HT-29 cells. As in macrophages [14], LT induced HDAC8 expression in both mRNA and the protein levels (Figure 1D). HDAC8-targeting siRNAs (si-HDAC8), but not random siRNAs (si-Random), significantly decreased the mRNA (Figure 1D, left panel) and protein (Figure 1D, right panel) levels of HDAC8 in both the parental and LT-resistant cells. Indeed, like PCI, si-HDAC8 prevented cell proliferation in LT-exposed HT-29 cells (Figure 1E).

### 3.2. LT-Induced Cell Cycle Arrest and Resistance Are Manifested by MEK1/2 Inhibition

**Figure 2:**
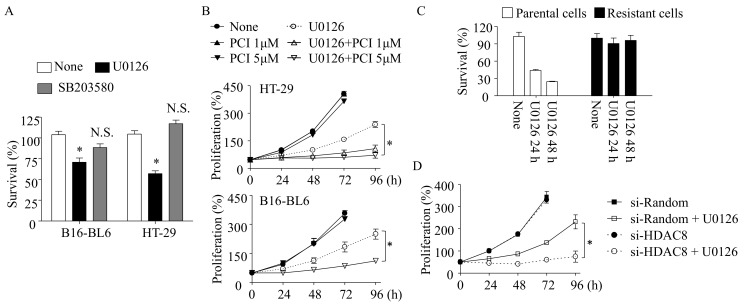
MEK1/2-ERK inhibition is involved in LT-induced cell cycle arrest in B16-BL6 and HT-29 cells. (**A**) B16-BL6 and HT-29 cells were seeded in 96-well plates for 18 h. Cells were then exposed to U0126 (12.5 µM) or SB203580 (12.5 µM) for 24 h, and cell survival was measured by the MTT assay. (**B**) Similarly, HT-29 (top panel) and B16-BL6 (bottom panel) cells were seeded in 96-well plates for 6 h. Cell were then treated with U0126 (12.5 µM) with or without PCI-34051 (PCI) for the time indicated. Cell proliferation was measured by the MTT assay. (**C**) Resistance HT-29 cells were selected after treating U0126 for 72 h. The surviving cells were replated and re-exposed to U0126 for the time indicated. Cell survival was measured by the MTT assay. (**D**) HT-29 cells were transfected with HDAC8 siRNA or random siRNA for 18 h and then treated with U0126 (12.5 µM) for the time indicated. Cell proliferation was measured by the MTT assay. (**A**–**D**) Data are expressed as means and ± SD (*n* = 3; N.S., not significant; *, *p* < 0.05 by the Student’s *t*-test).

LT inactivates both the MEK1/2-ERK and MEK3/6-p38 signaling pathways by cleaving MEK1-7, except MEK5 [24]. Therefore, we examined if these two signaling pathways were involved in preventing cell proliferation in LT-treated cells. In both B16-BL6 and HT-29 cells, the MEK1/2 inhibitor U0126, but not the p38 inhibitor SB203580, decreased cell survival to the similar extent observed by LT (Figure 1A and Figure 2A). In both B16-BL6 and HT-29 cells, the U0126 treatment also transiently inhibited cell proliferation for ~48 h, and cells started proliferating after 72 h of the treatment (Figure 2B). As in LT-resistant cells, when U0126-resistant cells were rechallenged with U0126, no defects in cell survival/proliferation were detected within 48 h (Figure 2C). PCI also prevented a resistance to U0126 in both HT-29 and B16-BL6 cells. Similar to LT-resistant cells, knocking down HDAC8 also prevented cell proliferation in U0126-exposed HT-29 cells (Figure 2D). These results suggest that the transient inhibition of cell proliferation and development of resistance induced by LT was mainly mediated by MEK1/2 inhibition.

### 3.3. Activation of AKT Is Mediated by HDAC8 and Required for Cell Proliferation in LT- and U0126-Resistant Cells

**Figure 3:**
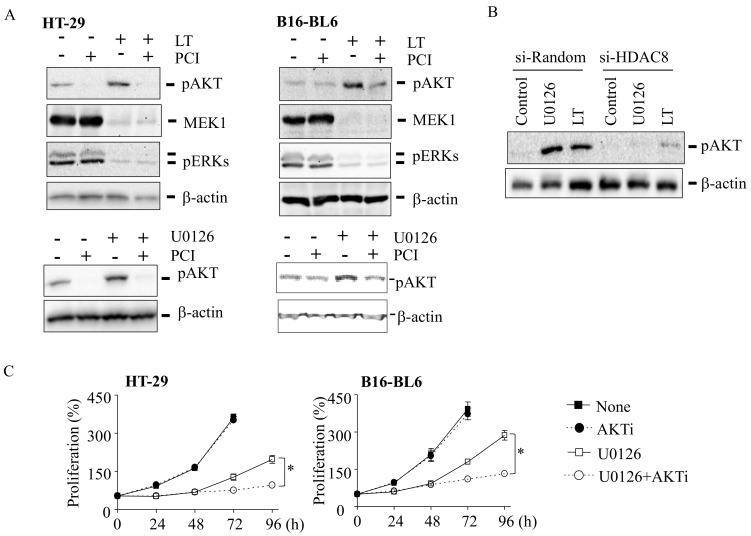
HDAC8 is required for AKT activation in LT- and U0126-resistant cells. (**A**) HT-29 and B16-BL6 cells were cultured with or without LT (500 ng/mL of each PA and LF, upper panel) or U0126 (12.5 µM, lower panel) together or without PCI-34051 (PCI: 5 µM) for 72 h. (**B**) HT-29 cells were transfected with random or HDAC8-targeting siRNAs for 18 h and then treated with LT (500 ng/mL of each PA and LF) or U0126 (12.5 µM) for 72 h. (A,B) AKT and ERK phosphorylation and MEK 1 cleavage were analyzed by Western blots. β-actin immunoblots were used for loading the controls. The Western blots shown are representative images of three independent experiments. (**C**) HT-29 and B16-BL6 cells were cultured in the presence or absence of U0126 (12.5 µM) for 48 h. Cells were further cultured with or without the AKT inhibitor (AKTi; 200 nM) for an additional 24 and 48 h. Cell proliferation was then measured by the MTT assay. Data are expressed as means and ± SD (*n* ≥ 3; *, *p* < 0.05 by the Student’s *t*-test).

In the absence of ERK activation, cell cycles can be proceeded by activating the AKT pathway [25,26]. Therefore, we examined whether LT and U0126 induced AKT activation and whether it was mediated by HDAC8. HT-29 and B16-BL6 cells were treated with LT or U0126 for 72 h, and the activation of AKT was examined by Western blots using an antibody specific for phosphorylated AKT at Ser-473 [27]. We first examined whether the MEK1/2-ERK signaling axis was inhibited in cells treated with LT for 72 h. Western blots using antibodies raised against the N-terminus (the first 12 amino acids) of MEK1 and phospho-specific ERK readily detected MEK1 and activated ERK in control and PCI-treated cells but not in LT- and LT + PCI-treated HT-29 and B16-BL6 cells (Figure 3A, upper panel). These data suggest that the MEK1/2-ERK signaling axis was inactivated in cells treated with LT for 72 h. In these cells, AKT was highly phosphorylated, which was prevented by PCI. Similarly, U0126-exposed cells showed a robust activation of AKT but not in PCI-exposed cells (Figure 3A, lower panels). Consistent with PCI, knocking down HDAC8 by siRNA also prevented LT- and U0126-induced AKT activation in HT-29 cells (Figure 3B). In addition, the inhibition of AKT by the AKT inhibitor (AKTi) prevented cell proliferation in U0126-resistant HT-29 and B16-BL6 cells (Figure 3C), suggesting that AKT activation was required for a resistance to cell cycle arrest. Altogether, these results suggest that HDAC8-mediated AKT activation is required for a resistance to LT and MEK1/2 inhibition.

### 3.4. HDAC8 is Involved in Regulating Expression of PLCB1 and DESC1 in MEK1/2 Inhibition-Resistant Cells

To examine the mechanism of HDAC8 in activating AKT, HT-29 cells were treated with LT in the presence or absence of PCI for 48 h, and the expression of over 47,000 transcripts were first examined using the Affymetrix microarray with GeneChip™ Human Genome U133 Plus 2.0 Array, followed by a gene ontology enrichment analysis using the Partek™ Genomics Suite^TM^. The microarray found ~1500 transcripts changed in expression more than two-fold by the LT or LT + PCI treatments. The top gene ontology enrichment was a cell cycle progress, followed by mitotic cell cycle process (Supplemental Table S1). A total of 141 cell cycle progress protein-coding genes were changed in expression by LT and/or LT + PCI (Supplemental Table S2). We expected that the genes involved in the LT resistance phenotype were likely upregulated in LT-resistant (LT-treated) cells but suppressed in re-sensitized (LT + PCI-treated) cells. Among the 141 genes, nine genes were upregulated in LT-resistant cells. Among the nine induced genes, PLCB1 (phospholipase C β1; also known as phosphatidylinositol-specific phospholipase C), which is involved in AKT activation [28], was downregulated in re-sensitized cells. We also examined the tumor suppressors that could be involved in the regulation of AKT activation and LT resistance. A total of 24 tumor-suppressor protein-coding genes were changed more than two-fold by LT or LT + PCI (Supplemental Table S3). We expected that the tumor suppressors involved in AKT inhibition were likely downregulated in LT-resistant cells but upregulated in LT re-sensitized cells. Among the 24 tumor suppressors, DESC1 (differentially expressed in squamous cell carcinoma 1), which is involved in the inhibition of AKT [29], was upregulated in LT re-sensitized cells. Therefore, we decided to further examine the roles of PLCB1 and DESC1 in AKT activation and resistance and re-sensitization to MEK1/2 inhibition.

**Figure 4:**
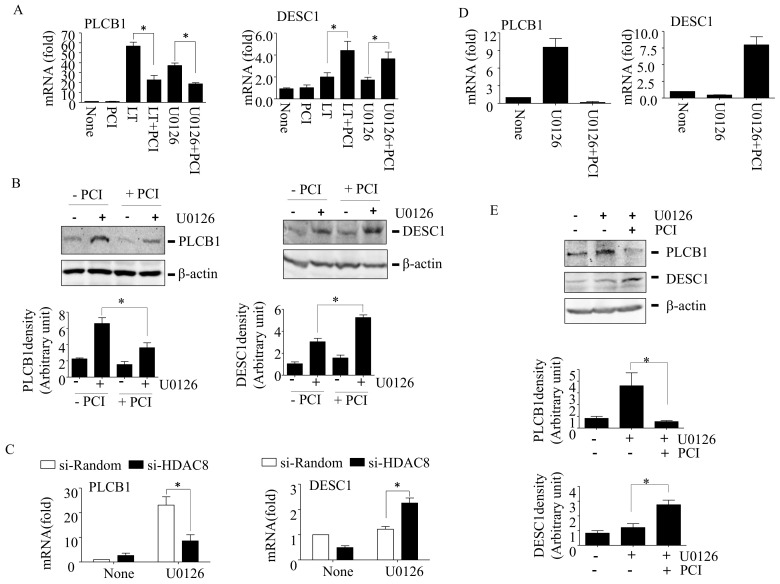
HDAC8 regulates the PLCB1 and DESC1 expression in LT- and U0126-treated HT-29 and B16-BL6 cells. (**A**) HT-29 cells were treated with none, LT (500 ng/mL of each PA and LF), or U0126 (12.5 µM) with or without PCI-34051 (PCI; 5 µM) for 48 h. The expression of PLCB1 and DESC1 mRNAs was measured by qPCR. (**B**) HT-29 cells were treated with none or U0126 with or without PCI for 48 h. The expression of PLCB1 and DESC1 were examined by Western blotting. The Western blots shown are representative images of two to three independent experiments. (**C**) HT-29 cells were transfected with random or HDAC8 siRNAs for 18 h. Cells were then treated with none or U0126 (12.5 µM) for 48 h, and the mRNA expression was analyzed by qPCR. (**D**,**E**) B16-BL6 cells were treated with U0126 (12.5 µM) with or without PCI-34051 (PCI; 5 µM) for 48 h. The expression of PLCB1 and DESC1 were examined by qPCR (**D**) and Western blotting (**E**). The Western blots shown are representative images of two to three independent experiments. Immunoblot intensities of the phospho-AKT bands were analyzed using Image Lab 6.0 (Bio-Rad; Hercules, CA, USA), and the relative band intensities were normalized to those of β-actin (**B**,**E**). The bar graph data are expressed as the means and ± SD (*n* = 2 to 3; *, *p* < 0.05 by the Student’s *t*-test).

We confirmed that LT and U0126 greatly enhanced the PLCB1 mRNA expression, which was significantly inhibited by PCI through quantitative (q)PCR analysis (Figure 4A, left panel). Consistent with the mRNA levels, the Western blot analysis also showed that U0126 alone induced PLCB1 expression, which was inhibited by PCI (Figure 4B, left panel). Furthermore, HDAC8-targeting siRNAs also recapitulated the effects of PCI, inhibiting and enhancing the expression of PLCB1 in U0126-exposed cells (Figure 4C, left panel). Similarly, LT and U0126, together with PCI, greatly enhanced the DESC1 expression in the mRNA (Figure 4A, right panel) and protein (Figure 4B, right panel) levels. Of note, U0126 alone slightly induced DESC1 in the protein levels but not in the mRNA levels. The discrepancy could be due to the transient upregulation and/or short half-life of DESC1 mRNAs. Additionally, HDAC8-targeting siRNAs had similar effects as PCI in inducing DESC1 expression in U0126-treated cells (Figure 4C, right panel). Like HT-29 cells, B16-BL6 cells also induced the expression of PLCB1 in response to U0126, which was inhibited by PCI, and the expression of DESC1 in response to U0126 + PCI in both the mRNA (Figure 4D) and protein (Figure 4E) levels. These results suggest that HDAC8 is involved in positively and negatively regulating the expression of PLCB1 and DESC1, respectively, in LT- and U0126-resistant cells.

### 3.5. Inhibition of PLCB1 Prevents Resistance to LT and MEK1/2 Inhibition in HT-29 Cells

**Figure 5:**
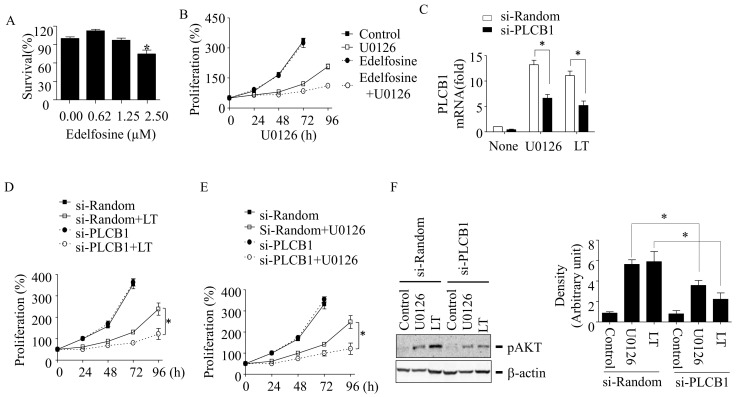
PLCB1 is required for resistance to LT and MEK1/2 inhibition in HT-29 cells. (**A**,**B**) Cells were seeded in 96-well plates and treated with various doses of edelfosine for 48 h (**A**) or treated with U0126 and/or edelfosine for the time indicated (**B**). Cell survival and proliferation were measured by the MTT assay. (**C**–**F**) Cells were transfected with random- or PLCB1-targeting siRNA (64 nM) for 18 h and then further cultured in the presence or absence of U0126 (12.5 µM) or LT (500 ng/mL of each PA and LF) for 72 h (**C**) or for the time indicated (**D**,**E**). The PLCB1 mRNA expression was analyzed by qPCR (**C**), and cell proliferation was measured by the MTT assay (**D**,**E**). Data are expressed as means and ± SD (*n* ≥ 3; *, *p* < 0.05 by the Student’s *t*-test). The AKT phosphorylation at Ser473 was analyzed in cells treated with U0126 or LT for 72 h by Western blotting ((**F**), left panel). The Western blots shown are representative of three independent experiments. Immunoblot intensities of the phospho-AKT bands were analyzed using Image Lab 6.0 (Bio-Rad; Hercules, CA, USA), and the relative band intensities were normalized to those of β-actin ((**F**). right panel). Data are expressed as means and ± SD (*n* = 3; *, *p* < 0.05 by the Student’s *t*-test).

To examine the role of PLCB1 in AKT activation and resistance to MEK1/2 inhibition, the effects of the PLCB1 inhibitor edelfosine [30] and PLCB1-targeting siRNAs were examined in LT- and/or U0126-resistant cells. Edelfosine at a noncytotoxic dose of 1.25 µM (Figure 5A) significantly inhibited cell proliferation in U0126-exposed cells (Figure 5B). Similarly, siRNA-targeting PLCB1 (si-PLCB1), which reduced the PLCB1 expression by 50% (Figure 5C), prevented cell proliferation in LT- and U0126-resistant HT-29 cells (Figure 5D,E). In line with these data, si-PLCB1 prevented AKT activation, which was induced by LT and U0126 (Figure 5F). These results suggest that PLCB1 is required for AKT activation and recovery from cell cycle arrest in LT- and U0126-exposed HT-29 cells.

### 3.6. DESC1 Prevents Resistance to LT and MEK1/2 Inhibition in HT-29 Cells

**Figure 6:**
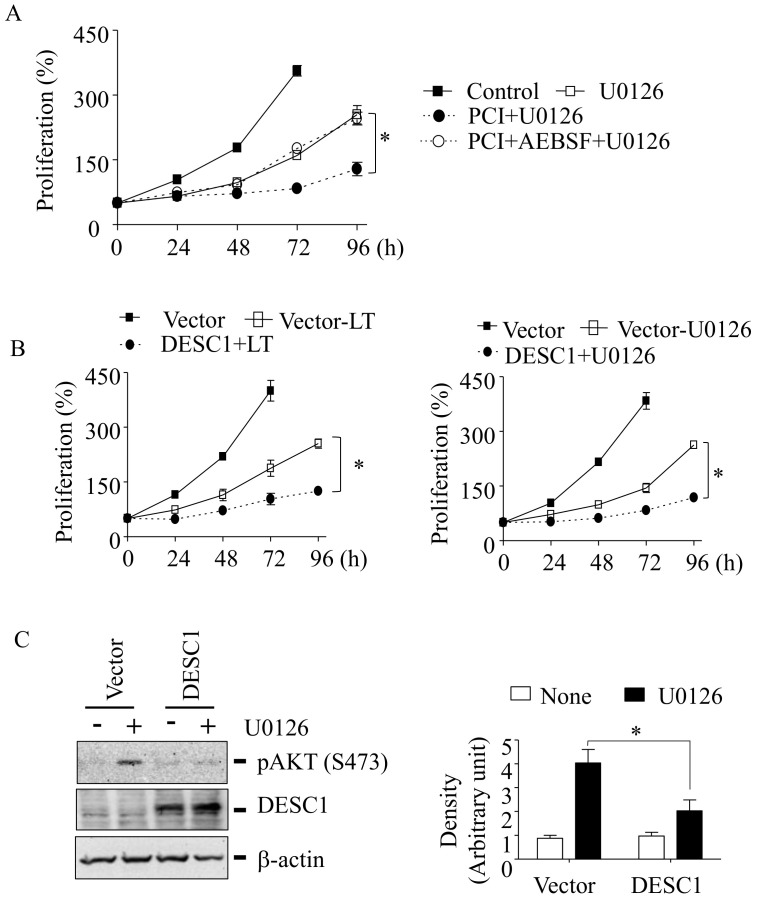
The high expression of DESC1 prevents AKT activation and resistance to LT and MEK1/2 inhibition in HT-29 cells. (**A**) HT-29 cells were plated in 96-well plates, as described above (Figure 5B) and cultured in the absence or presence of AEBSF or U0126 or PCI + U0126 or PCI + AEBSF + U0126 for the time indicated. Cell proliferation was measured by the MTT assay. Data are expressed as means and ± SD (*n* ≥ 3; *, *p* < 0.05 by the Student’s *t*-test). (**B**) HT-29 cells were transfected with a control vector or DESC1 plasmid using lipofectamine 2000 or lipofectamine 3000 for 18 h and replated to 96-well plates, followed by a treatment of LT (500-ng/mL PA or 500-ng/mL LF) or U0126 (12.5 µM) for the time indicated. Cell proliferation was measured by the MTT assay. (**C**) Similarly, HT-29 cells were transfected with the control vector or DESC1 plasmid. Cells were then cultured with or without U0126 (12.5 µM) for 72 h. AKT phosphorylation was analyzed by immunoblotting using the phospho-AKTser473 antibody (top blot in the left panel), and DESC1 overexpression was confirmed by Western blotting using DESC1 antibody (middle blot in the left panel). The immunoblot against β-actin was used for the loading control (bottom blot in the left panel). The results are representative blots from three independent experiments. The immunoreactivities against phospho-AKT were analyzed using Image Lab 6.0 (Bio-Rad; Hercules, CA, USA), and the relative immunoreactivity to phospho-AKT (Ser 473) was normalized to those of β-actin. Data are expressed as means and ± SD (*n* = 3; *, *p* < 0.05 by the Student’s *t*-test).

To examine the role of DESC1 in resistance to LT and MEK1/2 inhibition, we first examined the effects of the broad-spectrum serine protease inhibitor AEBSF, which inhibits cell membrane-associated proteases, including DESC1 [31]. AEBSF was able to reverse the PCI effect on the resistance to U0126 (Figure 6A), suggesting that the increase of DESC1 expression was involved in preventing the resistance to MEK1/2 inhibition. To further confirm its role in resistance, we ectopically expressed DESC1 and examined the resistance to LT and U0126. As expected, the overexpression of DESC1 mimicked the effect of PCI and inhibited the resistance to LT and U0126 in HT-29 cells (Figure 6B). Additionally, the overexpression of DESC1 inhibited AKT activation in U0126-resistant cells (Figure 6C). Altogether, these results suggest that the upregulation of DESC1 by PCI in U0126-resistant cells inhibits AKT activation and re-sensitized cells to MEK1/2 inhibition.

## 4. Discussion

The resistance of tumor cells to RAF/MEK inhibition is a transient and acquired adaptive response of the surviving cells due, in part, to the incomplete inactivation of ERK [2,3]. LT is a potential biological agent that effectively inhibits both MEK1/2-ERK and MEK3/6-p38 MAPK and, thus, is expected to render more pronounced effects on the cell cycle arrest and cell death [6]. The four cancer cell lines examined, harboring BRAF or NRAS mutations [6,20,21,22,23], were susceptible to LT (Figure 1A). Among the susceptible cells, MDA and SK-MEL human melanoma cells failed to develop a resistance to LT and, eventually, were all killed by LT (Figure 1B). Since MDA and SK-MEL cells develop a resistance to U0126 (data not shown) and BRAF inhibitors [6,18], but not LT (Figure 1B), it is possible that robust MEK1/2-ERK inhibition and/or the inhibition of both MEK1/2-ERK and MEK3/6-p38 signaling axes by LT prevented the development of resistance in these cells. Indeed, the incomplete inhibition of MEK1/2-ERK can lead to resistance due to residual or compensatory ERK activation [2,3,21]. These observations provided the theoretic grounds for combinatory therapies using both RAF and MEK inhibitors with positive outcomes. Unlike MDA and SK-MEL cells, murine melanoma B16-BL6 and human colorectal HT-29 cells still escaped from LT-induced MEK1/2-ERK inhibition (Figure 1B). In these cells, the MEK1/2 inhibitor U0126, but not the p38 inhibitor SB203580, mimicked LT cytotoxicity and the resistance profiles, suggesting that the inhibition of MEK1/2-ERK signaling is the main culprit of LT-induced cytotoxicity and activation of an alternative cell proliferation pathway(s).

Among the various cell survival pathways, the PI3K-AKT signaling axis can induce cell proliferation in the absence of MEK1/2-ERK signaling. In previous studies, MEK1/2-ERK inhibition also induces activation of the PI3K-AKT signaling axis and leads to resistance [25,32]. We also showed that human macrophages develop a resistance to LT-induced cell cycle arrest through activating the PI3K-AKT signal pathway [26]. The role of PI3K/AKT signaling in cancer cell proliferation and drug resistance has been well-documented [33]. Similarly, there are various potential mechanisms that AKT protects the cells from cell cycle arrest. We and others showed that inhibition of the glycogen synthase kinase 3β (GSK3b) by AKT (mediated by the S9 phosphorylation of GSK3b) is a key downstream event that protects the cells from cell cycle arrest [26], enhances cell proliferation [34], and promotes the resistance to various stresses [35]. However, GSK3b is a multifaceted enzyme targeting numerous protein substrates involved in both tumor cell growth and suppression [36]. Therefore, the involvement of GSK3b in the resistance to MEK1/2 inhibition warrants further studies.

Although, to date, the mechanism by which MEK1/2-ERK inhibition adaptively induces the PI3K-AKT signaling axis is not fully delineated, epigenetic reprogramming mediated by HDAC8 was shown to be involved in the resistance to MEK-ERK inhibitors [14,18]. In previous studies, we showed that HDAC8 suppresses the expression of the phosphatase-tensin homolog (PTEN; a negative regulator of PI3K) that enhances PI3K-AKT signaling in LT-resistant macrophages [14]. However, unlike in macrophages [14], LT and U0126 had no significant effects on PTEN expression in these cells (data not shown). In human melanoma, HDAC8 was also shown to induce a resistance to BRAF inhibition through targeting c-JUN [18]. In the study, HDAC8 directly deacetylates c-JUN at lysine 273, which enhances the transcriptional activation of receptor tyrosine kinases, such as EGFR, that induce a subsequent basal activation of ERK and AKT. Here, we found that the resistance to LT and U0126 also required HDAC8 in HT-29 and B16-BL6 cells (Figure 1, Figure 2 and Figure 3). Furthermore, we found that PLCB1 and DESC1 played key roles in the HDAC8-meidated resistance to MEK1/2 inhibition.

PLCB1 cleaves phosphatidylinositol 4,5-biphosphate and produces inositol 1,4,5-trisphosphate (IP3) and diacylglycerol (DAG). These second messengers activate PKCs and intracellular Ca^2+^ release in the cytoplasm [37]. PLCB1 also localizes in the nucleus, where it regulates transcription by releasing the second messengers and directly interacting with various nuclear proteins [38]. In various cells, the overexpression or activation of PLCB1 renders cell proliferation [39,40] and resistance to oxidative stresses [28,41] through activating PKC, ERK, and AKT and enhancing the expression of cyclin D3 and E. Here, we showed that PLCB1 was required for the resistance to LT and U0126 through, at least in part, by activating AKT in HT-29 cells (Figure 5). Further studies are required to unravel how PLCB1 leads to AKT activation and whether it depends on PKCs and/or Ca^2+^ release in the cytoplasm or nucleus. In addition, PLCB1 is activated by G-protein-coupled receptors (GPCRs), whose expression is one of the top-ranked protein classes associated with the resistance to MEK inhibitors in melanoma [42]. Therefore, it is possible that signaling from GPCRs could confer survival benefits for cells expressing high levels of PLCB1. The high expression of PLCB1 was also shown to be related to the development and poor prognosis of various cancers, including hepatocarcinoma [43,44,45], colorectal cancers [46,47], non-small cell lung carcinoma [45], breast cancer [48], and acute myeloid leukemia [49], suggesting its oncogenic role in different cancers.

DESC1 is a member of the type II transmembrane serine protease (also known as transmembrane protease, serine 11E; TMPRSS11E) and downregulated in squamous cell carcinoma of the head and neck [50] and esophageal squamous cell carcinoma [51]. DESC1 was demonstrated to be a tumor suppressor that cleaves EGFR and inhibits AKT activation that sensitizes cell death in esophageal squamous cells carcinoma [29,52]. Here, we also found that the ectopic overexpression of DESC1 inhibited AKT activation (Figure 6C) and prevented the development of a resistance to LT and U0126 (Figure 6B). In contrast, the serine protease inhibitor AEBSF prevented the effect of PCI in U0126-treated cells (Figure 6A). These results suggest that HDAC8 inhibition also, at least in part, re-sensitized HT-29 cells to MEK1/2-ERK inhibition through inducing DESC1 expression.

It is intriguing that HDAC8 renders a resistance by differently regulating the gene expression. Silencing the DESC1 expression by HDAC8 is anticipated, since HDAC8 deacetylates N-terminal tails of core histones and interacts with the corepressors [53,54]. Therefore, the inhibition of HDAC8 could lead to DESC1 transcription through targeting its cis-regulatory elements (promoter and enhancers). In addition, the DESC1 expression was shown to be regulated by the long non-coding RNA tumor-suppressor candidate 7 (TUSC7) that inactivates DESC1-targeting miR-224 [52], yet suggests an indirect regulation of DESC1 expression through noncoding RNAs. Further studies are needed to delineate the involvement of the cis-regulatory elements and/or TUSC7/miR-224 in regulating the DESC1 expression by HDAC8. Unlike DESCI, HDAC8 inhibition suppressed PLCB1 expression (Figure 4). It is possible that HDAC8 induces PLCB1 through deacetylating/activating c-JUN, as in the BRAF inhibitor-resistant melanoma [18]. However, the involvement of c-JUN in PLCB1 expression has yet to be established. PLCB1 expression is also controlled by miRs, such as miR-3184 in hepatocellular carcinoma [44] and miR-423-5p in glioblastoma cells [55]. Since HDAC8 can suppress the expression of certain miRs [56], PLCB1 could be induced by HDAC8 through the negative regulation of miRs. Delineating the downstream mechanisms of HDAC8 will reveal more specific targets in controlling resistance and warrants further studies.

**Figure 7:**
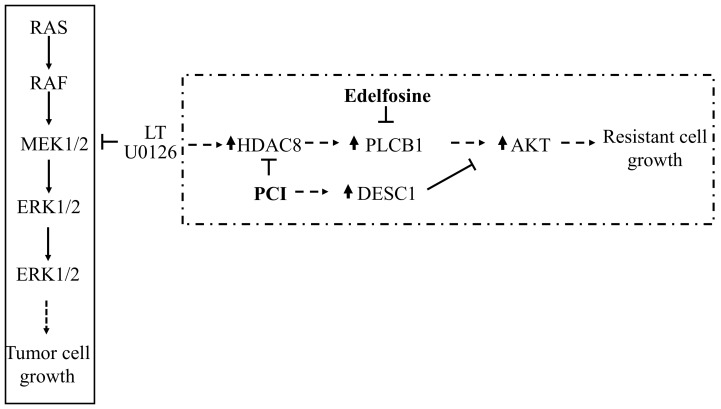
Schematic presentation of the signaling pathways for tumor cell growth and the resistance mechanism proposed. Mutations in NRAS and BRAF lead to tumor cell growth through activating the MEK1/2-ERK pathway. LT and U0126 inhibit MEK1/2 and prevent tumor cell growth (solid box). However, the inhibition of MEK1/2 by LT or U0126 can lead to an HDAC8-dependent cell proliferation pathway that activates AKT through enhancing the PLCB1 expression in a low-DESC1 background (dotted box). The inhibition of HDAC8 can lead to the suppression of PLCB1 expression and induction of DESC1 expression, both of which cooperatively inhibit AKT activation and a resistance to MEK1/2 inhibition.

In summary, MEK1/2 inhibitors or the biological agent LT, which can provide more potent and tumor-specific delivery, inhibit tumor cell growth by inhibiting the perpetually activated MEK1/2-ERK cell proliferation pathway (Figure 7, solid box). However, the inhibition of MEK1/2-ERK can lead to AKT activation through HDAC8 (Figure 7, dotted box). We found that HDAC8 induced the PLCB1 expression and subsequent AKT activation in low basal DESC1 expression/activity. The inhibition of HDAC8 prevented PLCB1 expression and, at the same time, increased DESC1 expression, both of which were involved in re-sensitizing cells to MEK1/2 inhibition. Therefore, targeting PLCB1 and DESC1 could be potential strategies for inhibiting the resistance to MEK1/2 inhibition in certain cancers with NRAS or BRAF mutations.

## Supplementary Materials

The following are available online at https://www.mdpi.com/article/10.3390/cells10051101/s1: Tables S1–S3: HT-29 cells were cultured with or without LT (500 ng/mL of each PA & LF) together with or without PCI-34051 (PCI: 5 μM) for 48 h. Total cellular RNAs were isolated using TRIzolTM (Ambion by Life Technologies; Carlsbad, CA, USA) and microarray was performed once using the GeneChip™ Human Genome U133 Plus 2.0 Array kit in the London Regional Genomics Centre (Robarts Research Institute in Western University, London, ON, Canada). Table S1: Gene ontology enrichment analysis was performed, and the top 10 gene ontology enrichment processes are provided, Table S2: Gene ontology enrichment analysis was performed, and the list of cell cycle progress-related genes changed more than 2-fold by LT or LT+PCI is provided. Highlighted genes are upregulated genes, Table S3: Gene ontology enrichment analysis was performed, and the list of tumor suppressor genes changed more than 2-fold by LT or LT+PCI is provided. Highlighted are genes upregulated by LT or LT+PCI.
