# Supplementary material for: HDAC8 Activates AKT through Upregulating PLCB1 and Suppressing DESC1 Expression in MEK1/2 Inhibition-Resistant Cells
Source: Cells. 2021 May 4;10(5):1101. doi: 10.3390/cells10051101 (PMC8147860; doi:10.3390/cells10051101)
Supplement: Supplementary file 1 [file cells-10-01101-s001.zip › cells-1200997-supplementary.pdf]

Supplemental Table S1: HT-29 cells were cultured with or without LT (500 ng/mL of each PA & LF) together or without PCI-34051 (PCI: 5 µM) for 48 h. Total cellular RNAs were isolated using TRIzolTM (Ambion by Life Technologies) and microarray was performed once using the GeneChip™ Human Genome U133 Plus 2.0 Array kit in the London Regional Genomics Centre (Robarts Research Institute in Western University, London, Ontario, CANADA). Gene ontology enrichment analysis was performed, and the top 10 gene ontology enrichment processes are provided.

function	type	Enrichment Score	Enrichment p-value
cell cycle process	biological process	101.293	1.02E-44
mitotic cell cycle process	biological process	95.3211	4.00E-42
chromosomal part	cellular component	56.3199	3.47E-25
chromosome organization	biological process	56.238	3.77E-25
cell division	biological process	51.2036	5.79E-23
nuclear part	cellular component	51.1509	6.10E-23
nuclear division	biological process	48.3014	1.05E-21
organelle fission	biological process	47.889	1.59E-21
nucleoplasm	cellular component	46.0864	9.66E-21
mitotic nuclear division	biological process	45.5635	1.63E-20

Supplemental Table S2: HT-29 cells were cultured with or without LT (500 ng/mL of each PA & LF) together or without PCI-34051 (PCI: 5 µM) for 48 h. Total cellular RNAs were isolated using TRIzolTM (Ambion by Life Technologies) and microarray was performed once using the GeneChip™ Human Genome U133 Plus 2.0 Array kit in the London Regional Genomics Centre (Robarts Research Institute in Western University, London, Ontario, CANADA). Gene ontology enrichment analysis was performed, and the list of cell cycle progress-related genes changed more than 2-fold by LT or LT+PCI is provided. Highlighted genes are upregulated genes.

Gene Symbol	RefSeq	Ratio(PCI+LT vs. CNT)	Ratio(LT vs. CNT)
RCC1	NM_001048194	0.285876	0.347751
CDCA8	NM_001256875	0.301431	0.364432
CDC20	NM_001255	0.202838	0.276023
KIF2C	NM_001297655	0.28741	0.412851
CDC7	NM_001134419	0.271888	0.365027
DCLRE1B	NM_022836	0.380033	0.461885
CKS1B	NM_001826	0.41233	0.457933

NUF2	NM_031423	0.366352	0.426331
RGS2	NM_002923	3.88544	3.06834
CENPF	NM_016343	0.370479	0.496498
ORC1	NM_001190818	0.408114	0.376708
IQGAP3	NM_178229	0.260466	0.363813
CENPL	NM_001127181	0.426725	0.534419
NEK2	NM_001204182	0.361652	0.358897
LIN9	NM_001270409	0.480212	0.558611
MCM10	NM_018518	0.144166	0.186486
MASTL	NM_001172303	0.49245	0.547641
CDK1	NM_001170406	0.278212	0.276161
ZWINT	NM_001005413	0.351716	0.391994
DNA2	NM_001080449	0.424627	0.536792
RRM1	NM_001033	0.454654	0.510663
FEN1	NM_004111	0.288035	0.32096
INCENP	NM_001040694	0.499674	0.557029
POLA2	NM_002689	0.391034	0.51125
KIF18A	NM_031217	0.28319	0.300318
NCAPD3	NM_015261	0.387565	0.412933
CDK2	NM_001290230	0.436831	0.517071
GAS2L3	NM_001303130	0.478251	0.485105
RFC5	NM_001130112	0.342727	0.421122
KNTC1	NM_014708	0.453629	0.525736
FOXM1	NM_001243088	0.197949	0.251364
PRIM1	NM_000946	0.390216	0.435871
APPL2	NM_001251904	2.37824	2.37824
POLE	NM_006231	0.489848	0.497844
RFC3	NM_002915	0.260031	0.292599
BORA	NM_001286746	0.41994	0.400214
CAB39L	NM_001079670	2.87853	2.8948
CDKN3	NM_001130851	0.237862	0.334401
VRK1	NM_003384	0.438171	0.473009
POLE2	NM_001197330	0.301611	0.297033
CASC5	NM_144508	0.363149	0.45838
HAUS2	NM_001130447	0.498951	0.507016
TUBGCP4	NM_001286414	0.434634	0.503921
CCNB2	NM_004701	0.249617	0.363116
ZWILCH	NM_001287821	0.286499	0.368202
KIF23	NM_001281301	0.316464	0.379613
TICRR	NM_001308025	0.287843	0.293981
CEP152	NM_001194998	0.482835	0.513506
CEP152	XM_011521374	0.481679	0.617719
CCNF	NM_001761	0.399187	0.472933

PLK1	NM_005030	0.197139	0.279959
ORC6	NM_014321	0.270574	0.331146
NUP93	NM_001242795	0.472603	0.503651
GINS3	NM_001126129	0.405069	0.527388
CENPN	NM_001100624	0.272596	0.317246
CDT1	NM_030928	0.301236	0.380926
GINS2	NM_016095	0.238456	0.238456
TUBG1	NM_001070	0.399215	0.416582
PRR11	NM_018304	0.199637	0.326401
BIRC5	NM_001012270	0.311819	0.311819
AURKB	NM_001256834	0.271242	0.344244
TOP2A	NM_001067	0.3539	0.441185
PSMC3IP	NM_001256014	0.402091	0.462407
TYMS	NM_001071	0.177862	0.259221
SEH1L	NM_001013437	0.368324	0.419993
RBBP8	NM_002894	0.498317	0.451902
SKA1	NM_001039535	0.282695	0.340353
CCNE1	NM_001238	0.342748	0.34046
PDCD2L	NM_032346	0.460542	0.535774
SPC24	NM_182513	0.303056	0.377067
RRM2	NM_001034	0.300244	0.34095
CENPA	NM_001042426	0.468281	0.586728
RPS27A	NM_001135592	0.445488	0.614994
NCAPH	NM_001281710	0.224695	0.283091
PPM1G	NM_177983	0.443257	0.525855
BUB1	NM_001278616	0.227497	0.3303
MCM6	NM_005915	0.320284	0.352843
SPC25	NM_020675	0.315408	0.381822
HJURP	NM_001282962	0.371331	0.432026
MCM8	NM_001281520	0.427451	0.493156
PLCB1	NM_015192	2.97299	4.1232
GINS1	NM_021067	0.276277	0.369938
MYBL2	NM_001278610	0.229278	0.295507
UBE2C	NM_001281741	0.304198	0.328864
PCNA	NM_002592	0.261789	0.309554
E2F1	NM_005225	0.295804	0.299625
RBL1	NM_002895	0.45033	0.484462
AURKA	NM_003600	0.342102	0.379798
CDC45	NM_001178010	0.309178	0.33773
MCM5	NM_006739	0.227065	0.292042
GTSE1	NM_016426	0.322018	0.412089
RANGAP1	NM_001278651	0.467146	0.668615
CENPM	NM_001002876	0.402886	0.422057

MCM2	NM_004526	0.334439	0.39631
SMC4	NM_001002800	0.317317	0.380391
SGOL1	NM_001012409	0.244391	0.321495
CDC25A	NM_001789	0.26076	0.240049
NCAPG	NM_022346	0.182977	0.283118
CCNG2	NM_004354	2.18542	2.18542
PLK4	NM_001190799	0.330495	0.382655
MND1	NM_001253861	0.268424	0.334273
PPAT	NM_002703	0.364533	0.368884
CENPE	NM_001286734	0.32225	0.44794
MAD2L1	NM_002358	0.214296	0.311943
CCNA2	NM_001237	0.188092	0.259087
CENPU	NM_024629	0.449788	0.449788
PELO	NM_015946	2.00204	2.00204
CCNB1	NM_031966	0.192794	0.269016
CENPH	NM_022909	0.403698	0.430568
KIF20A	NM_005733	0.400406	0.445795
NUP155	NM_001278312	0.482291	0.521369
CENPK	NM_001267038	0.243588	0.284678
CDC25C	NM_001287582	0.48917	0.516851
GMNN	NM_001251989	0.379135	0.470974
HIST1H4C	NM_003542	0.449769	0.482229
CENPQ	NM_018132	0.439105	0.526422
CENPW	NM_001012507	0.247306	0.326343
HIST1H4L	NM_003546	0.312235	0.3152
MCM3	NM_001270472	0.291034	0.324251
FBXO5	NM_001142522	0.340748	0.426026
DBF4	NM_006716	0.240242	0.272895
AKAP9	NM_005751	2.41764	2.42304
NUP205	NM_015135	0.445218	0.473976
TBRG4	NM_001261834	0.426676	0.508931
RFC2	NM_001278791	0.328773	0.331529
MCM7	NM_001278595	0.469683	0.605183
XRCC2	NM_005431	0.266924	0.323332
NCAPG2	NM_001281932	0.417351	0.438126
ESCO2	NM_001017420	0.28137	0.367127
MCM4	NM_005914	0.244048	0.284462
MELK	NM_001256685	0.261445	0.295467
CENPP	NM_001012267	0.475247	0.548013
SMC2	NM_001042550	0.468408	0.530469
NUP188	NM_015354	0.49515	0.597079
TUBB4B	NM_006088	0.494319	0.487004
CDKN2B	NM_004936	2.20441	1.88372

SDCCAG3	NM_001039707	0.413875	0.545392
POLA1	NM_016937	0.450899	0.574821
RRAGB	NM_006064	2.0481	2.11187
CENPI	NM_006733	0.39795	0.475615
ERCC6L	NM_017669	0.474085	0.512131

Supplemental Table S3: HT-29 cells were cultured with or without LT (500 ng/mL of each PA & LF) together or without PCI-34051 (PCI: 5 µM) for 48 h. Total cellular RNAs were isolated using TRIzolTM (Ambion by Life Technologies) and microarray was performed once using the GeneChip™ Human Genome U133 Plus 2.0 Array kit in the London Regional Genomics Centre (Robarts Research Institute in Western University, London, Ontario, CANADA). Gene ontology enrichment analysis was performed, and the list of tumor suppressor genes changed more than 2-fold by LT or LT+PCI is provided. Highlighted are genes upregulated by LT or LT+PCI.

		LT vs. CNT	PCI+LT vs. CNT
NM_006763	BTG2	6.09	4.3
NM_004562	PARK2	2.01	2.13
NM_001423	EMP1	0.32	0.36
NM_001313972	TXNIP	4	4.64
NM_001134419	CDC7	0.36	0.27
NM_001270409	LIN9	0.56	0.48
NM_001290230	CDK2	0.52	0.43
NM_004936	CDKN2B	1.88	2.2
NM01224	DKK1	0.22	0.18
NM_001199492	PDCD4	3.57	3.73
NM_001080521	RASSF10	5.05	5.75
NM_001114121	CHEK1	0.41	0.35
NM_001003940	BMF	4.35	4.99
NM_016639	TNFRSF12A	0.23	0.2
NM_002894	RBBP8	0.45	0.5
NM_001190942	TNFRSF10	2.89	3.17
NM_003897	IER3	0.42	0.45
NM_001244262	HBP1	2.48	2.47
NM_001039372	HEPACAM	10.7	8.44
NM_001135733	TP53INP1	6.94	6.46
NM_001242463	FBXO32	4.39	3.43
NM_001288729	DAPK1	2.75	3.02

NM_014058	DESC1(TMPRSS11E)	1	2.03
NM_018191	RCBTB1	2.13	2.098
